# Smartphone and App Usage in Orthopedics and Trauma Surgery: Survey Study of Physicians Regarding Acceptance, Risks, and Future Prospects in Germany

**DOI:** 10.2196/14787

**Published:** 2020-11-30

**Authors:** Florian Dittrich, David Alexander Back, Anna Katharina Harren, Stefan Landgraeber, Felix Reinecke, Sebastian Serong, Sascha Beck

**Affiliations:** 1 Department for Orthopaedics and Orthopaedic Surgery Saarland University Medical Center and Saarland University Faculty of Medicine Homburg Germany; 2 Joint Centre Bergisch Land Department for Orthopaedics Sana Fabricius Clinic Remscheid Remscheid Germany; 3 Clinic of Traumatology and Orthopedics Bundeswehr Hospital Berlin Germany; 4 Department of Plastic, Reconstructive & Aesthetic Surgery Specialized Clinic Hornheide Münster Germany; 5 Clinic of Trauma, Hand and Reconstructive Surgery Essen University Hospital Essen Germany; 6 Clinic for Orthopaedics and Trauma Surgery Sportsclinic Hellersen Lüdenscheid Germany

**Keywords:** mHealth, smartphone, communication, medicine, surveys and questionnaires, technology, orthopedics, trauma surgery

## Abstract

**Background:**

In the course of digitization, smartphones are affecting an increasing number of areas of users’ lives, giving them almost ubiquitous access to the internet and other web applications. Mobile health (mHealth) has become an integral part of some areas of patient care. In contrast to other disciplines, routine integration of mobile devices in orthopedics and trauma surgery in Germany is still in its infancy.

**Objective:**

This study aimed to investigate physicians’ current state of opinion regarding acceptance, future prospects, and risks of medical apps in the field of orthopedics and trauma surgery in Germany.

**Methods:**

A web-based survey among orthopedics and trauma surgeons in German university hospitals on the use of medical apps in everyday clinical practice was conducted between September 2018 and February 2019. The survey consisted of 13 open- and closed-ended or multiple-choice questions. A logistic regression analysis was performed to ascertain the effects of interindividual characteristics on the likelihood of participants’ app and smartphone usage behavior.

**Results:**

A total of 206 physicians participated in the survey. All of the participants (206/206, 100%) owned a smartphone, and 79.1% (159/201) used the device, while 64.7% (130/201) used apps regularly in everyday clinical practice. Medical apps were perceived as beneficial, given their substantial future promise, by 90.1% (181/201) of the participants. However, 62.5% (120/192) of the participants were not satisfied with the current supply of medical apps in app stores. Desired specifications for future apps were “intuitive usability” (167/201, 83.1%), “no advertising” (145/201, 72.1%), and “free apps” (92/201, 45.8%). The attributes “transparent app development and app sponsoring” (75/201, 37.3%) and the existence of an “easy-to-understand privacy statement” (50/201, 24.9%) were of minor relevance. The majority of the participants (162/194, 83.5%) considered that future apps in the field of “medical research” would provide the greatest benefit. The greatest predicted risks were “data misuse” (147/189, 77.8%), “usage of untrustworthy apps” (135/189, 71.4%), and “alienation from patients” (51/189, 27.0%). Increasing age was significantly associated with a reduction in the likelihood of regular smartphone (odds ratio [OR] 0.91, 95% CI 0.86-0.97; *P*=.002) and app (OR 0.90, 95% CI 0.85-0.96; *P*=.001) usage, while the medical profession grade had no significant impact on the usage behavior.

**Conclusions:**

The study demonstrates that young German doctors in orthopedics and trauma surgery already use smartphones and apps in everyday clinical practice. Medical apps are considered to play an important role in the future. However, a significant discrepancy exists between the supply and demand of mHealth applications, which creates a legal and ethical vacuum with regard to data protection.

## Introduction

The “digital transformation” of medicine began about 30 years ago with the replacement of analogue recordings by electronic systems in clinics and outpatient care facilities. At present, the establishment of the electronic health card to record patient data dominates health policy debates on the subject of digitization in Germany [1]. Since the beginning of the 21st century, information technology has not only served automation and optimization, but has also led to automation and individualization of processes by using “disruptive technologies.” However, the digital transformation goes far beyond the literal digital conversion of analogue media [2]. It stands for a dynamic reformation based on digital technologies that include society as a whole, the health care system with its involved companies, treatment facilities, and health care professions [3].

Smartphones, which have been rapidly integrated into various areas of life, have replaced mobile phones with keypads within a very short time, and thus, they can be classified as a disruptive technology [4]. The omnipresent mobile device offers qualitatively different functions in addition to pure web-based benefits via the implementation of native application programming interfaces and operating system functions. The portability and accessibility of smartphones enable their usage anywhere and at any time [5]. Numerous studies have shown that the number of smartphone users and the time spent using smartphones per day continue to rise significantly [6-8]. Interestingly, 81% of Germany’s 62 million internet users used smartphones to access the internet in 2016 [9]. The rise in smartphone usage and the introduction of app stores have facilitated individually customized software installation. This ready individualization provides people almost ubiquitous access to the vast amounts of available web-based information as well as innovative technologies accessible through mobile web-based applications [10].

The term “mobile application,” colloquially known as “app,” is defined as application software for mobile operating systems. Apps enable web-based, application-specific functions by operating with an intuitive user interface (“frontend”). The daily use of apps in private and professional contexts, such as online banking or communication via messenger and email, has become commonplace [11]. However, the use and integration of smartphones in medical care in Germany, especially in the fields of orthopedics and trauma surgery, is still in its infancy.

Although a total of 39,319 apps appeared under the “Medical” category on Google Play Store (Google LLC) in March 2019 [12], currently, no unified definition of “apps” exists in the medical context; they may be called “lifestyle apps,” “health apps,” “care apps,” or “medical apps.” Apps developed for patients should be differentiated from those directed at medical staff and those with a direct influence on the diagnosis of or therapy for a disease, and are therefore to be regarded as medical devices, unlike apps that focus purely on lifestyle [13].

Simple distribution, typically low development costs, and ease of use have resulted in a constantly growing and unmanageable supply of apps. Moreover, the offers provided by app stores are often unclear and heterogeneous for their users due to their complexity, dynamics, and rudimentarily regulated organization [14]. The range of available apps is so dynamic that their quantity and quality can vary even from day to day [14,15].

Irrespective of the rapid developments in the field of medical apps, young physicians especially have experienced a fundamental change in their information behavior over the past few years [16]. An increasing number of studies have focused on the benefits and consequences of smartphone use in the fields of orthopedics and trauma surgery [17-22]. Today, the majority of American orthopedic surgeons use smartphones and a wide variety of apps in their daily clinical work [23]. Apps published by representative institutions such as the AO Foundation, those focusing on education, and apps serving as a reference demonstrate the highest usage rates among orthopedic surgeons in Germany. However, the number of regularly used apps is low. The causes of this lack of acceptance have not yet been clarified conclusively [24].

Thus, the integration and use of smartphones in medical care, especially in the fields of orthopedics and trauma surgery, are in the nascent stage. In view of the rising smartphone usage in highly developed societies, this study aimed to investigate German physicians’ current opinion regarding their acceptance, future prospects, and risks of use in the field of orthopedics and trauma surgery in Germany.

## Methods

### Study Design and Sample

A survey among 836 orthopedics and trauma surgeons in German university hospitals on the use of medical apps in everyday clinical practice was conducted between September 2018 and February 2019. The link for the digital questionnaire, which appeared on a Google Docs platform (Google LLC), was sent to the participants by email. The email addresses of the potential test persons were generated manually via the homepages of the university hospitals or established in-house email distribution lists. The participants’ profession (orthopedic or trauma surgery) was confirmed before starting the survey. A positive vote from the responsible ethics committee was obtained in advance (No. 18-8142-BO). All of the investigations described in this study were carried out with the consent of the abovementioned committee and in accordance with national law and the Declaration of Helsinki.

### Survey Items

The survey consisted of 13 open- and closed-ended or multiple-choice questions encompassing the following domains: (1) The medical profession, qualification, and age of each participant; (2) their usage behavior regarding smartphones and medical apps; (3) a subjective assessment of currently available medical apps, and future potential risks and benefits; and (4) an evaluation of the readiness to purchase apps.

As no gold standard exists for mHealth surveys, our research group developed app- and smartphone-related questions. The questions were validated by a group of medical experts in the field of digitization and survey development, and their feedback was integrated into the final draft of the questionnaire. Their medical competence was determined by the level of training and years of clinical practice. According to our interpretation, a senior or chief physician has higher medical competence than a resident physician. The survey participants did not have to answer every question; they could skip parts of the questionnaire. The participants completed the survey within 90 seconds on average. Medical apps of relevance for this study were defined as apps with a clear medical purpose. Messenger services or system apps, such as WhatsApp (WhatsApp Ireland Ltd.) or a browser, were not taken into consideration.

### Data Analysis

The survey results were temporarily saved on the web in a Google Drive folder (Google LLC) and then transferred into an Excel table (Microsoft Corp.). Descriptive statistics were calculated for all items. A logistic regression analysis was performed to ascertain the effects of age and medical qualification grade on the likelihood of app and smartphone usage by the participating orthopedics or trauma surgeons and their usage behavior. Equivalent to the t test in linear regression, the Wald test checks the null hypothesis that the respective regression coefficient B in the population takes the value 0. Statistical significance was determined by *P* values less than .05. All statistical analyses were conducted using SPSS (version 25, IBM Corp.).

## Results

### Descriptive Statistics

In total, 24.6% (206/836) of the contacted orthopedic or trauma surgeons participated in the survey. Tables 1 and 2 show the characteristics of the survey participants as opposed to those of the members of the German Society for Orthopaedics and Trauma (DGOU).

**Table 1:** Characteristics of the DGOU^a^ members (N=10,487) and survey respondents (N=189) with regard to age.

Age groups (years)		Values
**DGOU, n (%)**		
	<35	1413 (13.47)
	36-45	3091 (29.47)
	46-55	2644 (25.21)
	56-65	1937 (18.47)
	>65	1402 (13.37)
**Survey respondents, n (%)**		
	<30	30 (15.87)
	30-39	93 (49.21)
	40-49	41 (21.69)
	50-59	16 (8.47)
	>60	9 (4.76)

^a^DGOU: Society for Orthopaedics and Trauma. Status as of February 2020. Source: DGOU Office.

**Table 2:** Characteristics of the DGOU^a^ members (N=10,487) and survey respondents (N=206) with regard to area of activity.

Area of activity	DGOU, n (%)	Survey respondents, n (%)
Higher education	1624 (15.49)	0 (0)
Resident physician	2686 (25.61)	93 (45.15)
Consultant	1093 (10.42)	41 (19.90)
Senior consultant	2932 (27.96)	67 (32.52)
Other medical employment	1702 (16.23)	5 (2.42)

^a^DGOU: Society for Orthopaedics and Trauma. Status as of February 2020. Source: DGOU Office.

**Figure 1:**
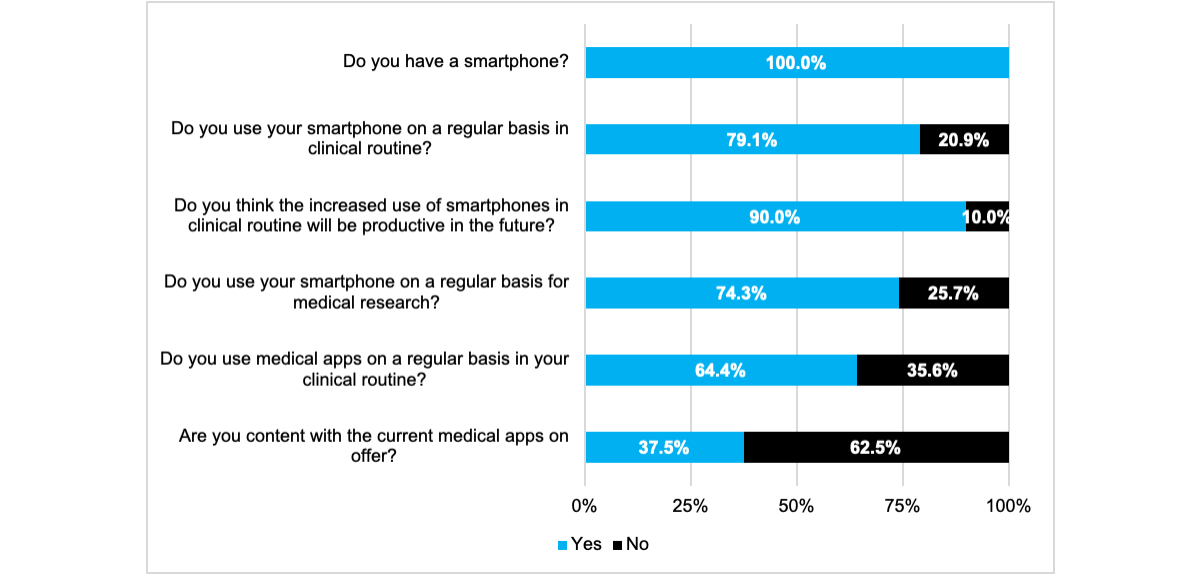
Usage behavior of smartphones and medical apps in orthopedics and trauma surgery (N=206).

All the participants stated that they owned a smartphone (206/206, 100%). Smartphones (159/201, 79.1%) and apps (130/201, 64.7%) were used regularly by the majority of doctors in their daily clinical routine and for medical research. Most surgeons were not satisfied with the current supply of medical apps in the app stores (120/192, 62.5%), but they felt that the use of smartphones on a regular basis offered great potential for the future of health care (181/201, 90.1%) (Figure 1).

**Figure 2:**
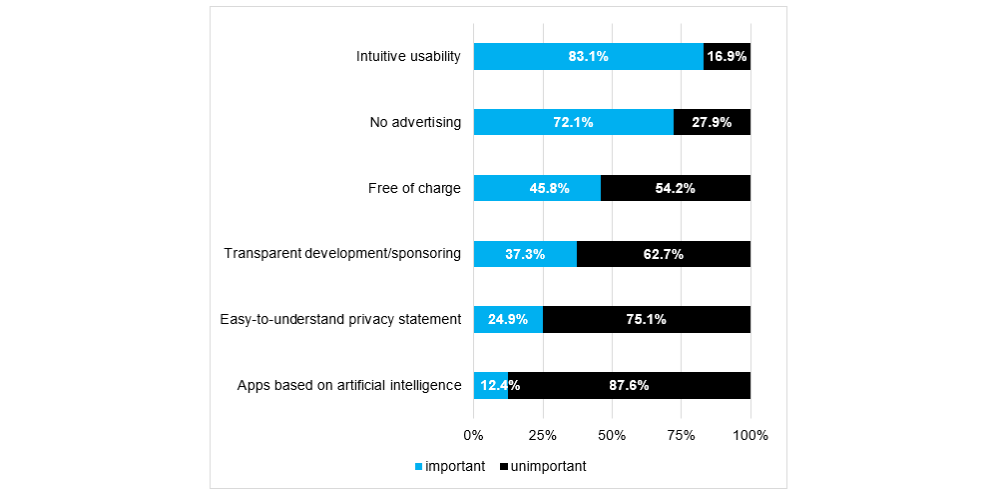
Functions or features important to the survey participants in future medical apps (indicated via responses to multiple-choice questions; N=194).

**Figure 3:**
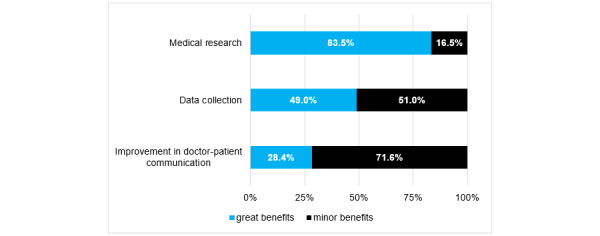
Participants’ responses regarding the greatest benefits of using of medical apps (indicated via responses to multiple-choice questions; N=201).

**Figure 4:**
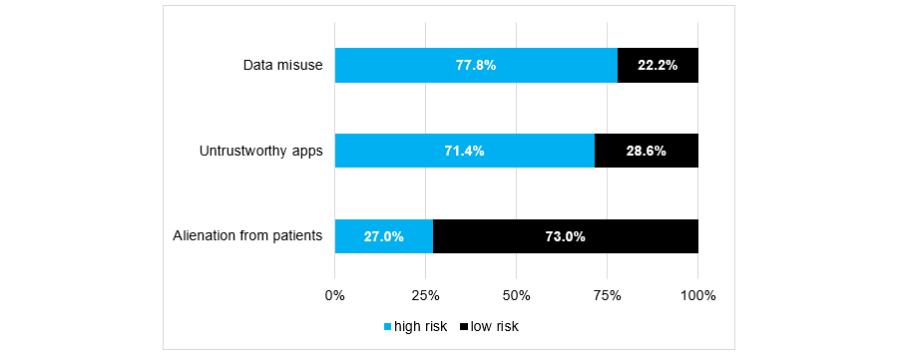
Participants’ responses regarding the risks that can arise from the regular use of medical apps in the future (indicated via responses to multiple-choice questions; N=189).

By analyzing the participants’ responses to the multiple-choice questions focused on the future potential, fields of application, and risks of day to day use of smartphones and apps in daily clinical routine, we were able to identify the most frequently requested features (Figure 2) as well as the greatest perceived benefits (Figure 3) and risks (Figure 4) of future app usage.

### Logistic Regression Analysis

Respondents who stated that smartphone usage has great potential for the future were 45.22 (*P*=.001) times more likely to use smartphones and 10.32 (*P*=.009) times more likely to use apps in their daily clinical routine than physicians who rejected the use of smartphones in everyday clinical practice. In addition, an increasing readiness to buy apps was associated with an increased likelihood of smartphone usage in daily clinical routine (odds ratio (OR) 1.73, 95% CI 1.32-2.26; *P*=.008). Increasing age demonstrated a significant negative correlation to regular smartphone (OR 0.91, 95% CI 0.86-0.97; *P*=.002) and app (OR 0.90, 95% CI 0.85-0.96; *P*=.001) usage. Increasing medical competence was accompanied by a decrease in the use of smartphones (*P*=.21) or apps (*P*=.58), but the result was not significant. The dissatisfaction with the current supply of apps was not associated with a significant correlation to app usage behavior (Tables 3 and 4). Conversely, none of the elicited parameters had a significant influence on satisfaction with the currently available apps on offer (data not shown).

**Table 3:** Statistical analysis of respondents’ answers to the question “Do you use your smartphone on a regular basis in your clinical routine?”^a^

	B	SE	Wald (*df*)	P	Exp (B)
Medical qualification	–0.42	0.33	1.60 (1)	.21	0.66
Age^b^	–0.09	0.03	9.16 (1)	.002	0.91
Future potential^b^	3.81	1.12	11.67 (1)	.001	45.22
App costs^b^	0.54	0.20	7.06 (1)	.01	1.72
Constant	0.69	1.49	0.22 (1)	.64	2.00

^a^χ^2^_4_=73.4; *P*<.001. The model explains 52.3% (Nagelkerke R^2^) of the variance and correctly classifies 88.2% of the cases.

^b^Values are significant for age, future potential, and app costs at *P*<.002, *P*<.001, and *P*<.01, respectively.

**Table 4:** Statistical analysis of respondents’ answers to the question “Do you use medical apps on a regular basis in your clinical routine?”^a^

	B	SE	Wald (*df*)	P	Exp (B)
Medical qualification	–0.15	0.27	0.31 (1)	.58	0.86
Age^b^	–0.10	0.03	12.00 (1)	.001	0.90
Future potential^b^	2.33	0.89	6.87 (1)	.011	10.32
App costs	0.26	0.16	2.56 (1)	.11	1.30
Apps offered	0.62	0.44	1.98 (1)	.16	1.86
Constant	1.63	1.32	1.53 (1)	.22	5.12

^a^χ^2^_4_=51.4; *P*<.001. The model explains 36.6% (Nagelkerke R^2^) of the variance and correctly classifies 76.9% of cases.

^b^Values are significant for age and future potential at *P*<.001 and *P*<.01, respectively.

The benefits of increased smartphone use in the future were not significantly influenced by age (*P*=.96), medical profession (*P*=.92), satisfaction with the currently available apps on offer (*P*=.13), and current app usage behavior (*P*=.73). However, the respondents who were already using their smartphones regularly in daily clinical practice (OR 150.3, 95% CI 5.04-4484.02; *P*=.004) and spent considerable amounts of money on apps (OR 2.03, 95% CI 1.13-3.62; *P*=.02) believed that the increased use of smartphones in clinical practice offered great potential. These parameters were significantly correlated (*P*=.004 and *P*=.02; Table 5). Older and experienced physicians demonstrated reduced usage of smartphones in the context of medical research (OR 0.93, 95% CI 0.89-0.97; *P*=.001) compared to their younger colleagues (Table 6).

**Table 5:** Statistical analysis of respondents’ answers to the question “Do you think the increased use of smartphones in clinical routine will be productive in the future?”^a^

	B	SE	Wald (*df*)	*P*	Exp(B)
Medical qualification	–0.06	0.56	0.01 (1)	.92	0.94
Age	–0.00	0.05	0.00 (1)	.96	1.00
App costs^b^	0.71	0.30	5.67 (1)	.02	2.03
App offer	–1.30	0.87	2.24 (1)	.13	0.27
Clinical smartphone usage^b^	5.01	1.73	8.37 (1)	.004	150.30
Clinical app usage	–0.52	1.49	0.12 (1)	.73	0.59
Constant	–0.91	2.74	0.11 (1)	.74	0.40

^a^χ^2^_4_=60.4; *P*<.001. The model explains 67.0% (Nagelkerke R^2^) of the variance and correctly classifies 95.2% of the cases.

^b^Values are significant for app costs and clinical smartphone usage at *P*<.02 and *P*<.004, respectively.

**Table 6:** Statistical analysis of respondents’ answers to the question “Do you use your smartphone on a regular basis for medical research?”^a^

	B	SE	Wald (*df*)	*P*	Exp (B)
Medical qualification	–0.09	0.24	0.14 (1)	.71	0.91
Age^b^	–0.08	0.02	11.78 (1)	.001	0.93
Constant	4.26	0.77	31.03 (1)	.001	71.05

^a^χ^2^_4_=21.5; *P*<.001. The model explains 16.2% (Nagelkerke R^2^) of the variance and correctly classifies 76.2% of the cases.

^b^Values are significant for age at *P*<.001.

## Discussion

### Principal Findings

The results of this study indicate that younger and less experienced doctors in the field of orthopedics and trauma surgery already use their smartphones and apps on a regular basis in clinical practice. Free apps and intuitive usability were considered to be the most important factors for regular app usage. In contrast, satisfaction with the currently available apps on offer was low. The greatest perceived risks regarding the use of mHealth apps were data misuse and the danger of using untrustworthy apps.

### Usage Behavior

Several important findings can be drawn from this study. First, smartphones and apps were used regularly by most of the participants (159/201, 79.1%; 130/201, 64.7) in their daily clinical routine. In the future, it is expected that these devices will not be confined to a minority (eg, medical students or young physicians); rather, they will commonly be utilized by all medical professionals. In detail, the physician’s age had a significant influence on the intensity of smartphone and app usage in clinical daily life. Younger physicians were more likely to integrate smartphones or apps into their daily work. Interestingly, medical qualifications did not have a significant influence in this regard. Although the use of digital media tended to decline with increasing medical qualification, even experienced doctors used smartphones in daily clinical routine. This result may be attributed to the very extensive range of apps supplied and the vast application possibilities of smartphones [25]. The different features offered cover the needs of a large target group. Overall, young doctors are more likely to use mobile devices, which can be explained by learned “media adaptation.” If people were influenced by the digital media in their adolescence, the use of proven and learned informational or communication tools and the corresponding behaviors are to be expected [26]. This aspect emphasizes the fact that young age, not lower medical qualification, is associated with frequent use of smartphones to carry out medical research, a finding that coincides with those of studies from other disciplines [3].

Current established user scenarios of apps in clinical practice focus on areas of education and reference purposes (Table 5). The number of orthopedics and trauma surgeons satisfied with the currently available apps on offer was quite low (72/192, 37.5%). The discrepancy between the high digital acceptance and the dissatisfaction with existing apps could lead to the use of inadequate apps in a medical context. For instance, the messenger service WhatsApp is commonly used as communication medium in everyday clinical practice. The benefits of a messenger service-based communication of text, image, and sound are evident, as it allows optimized professional consultations in comparison to simple verbal conversations by telephone. However, the clinical use of WhatsApp in a medical treatment context is problematic from the ethical and legal points of view (ie, data protection) and may lead to a violation of medical confidentiality [27,28].

Improper app usage in everyday clinical practice could give rise to problems if the content-related algorithms and guidelines used do not comply with the applicable country-level requirements. In addition to these non-negligible content-specific discrepancies, the variations in software specifications across countries must be taken into account.

Apps that exclude any user-related liability (eg, by the statement “only for training purposes”) are also questionable in the context of Germany’s Act on Medical Devices [29]. Nevertheless, there is reason to believe that these apps are used in clinical practice contexts [30]. Although such apps may contain information relevant to clinical practice, thus presenting an objectifiable added value for the user, in the event of damage, accusations of culpability stemming from the use of a (potentially) unsuitable/ Conformité Européenne (CE)-/Food and Drug Administration-certified app in the treatment context must be refuted. The surgeon cannot assume that every app is free of errors. Therefore, before using the app, physicians must assure themselves that the app is suitable for the intended purpose and is also recognizably safe in order to be covered by liability law [31]. This aspect illustrates the demand for an objective and transparent evaluation process for medical apps to protect doctors and hospitals against liability consequences. Textbox 1 shows established medical apps recommended or evaluated positively by German orthopedic or trauma surgeons [24,32].

**Established medical apps recommended or evaluated positively by German orthopedic or trauma surgeons.**

**Apps for referencing**

- *Surgical training*: AO Surgery Reference, AO/OTA Fracture Classification, Touch Surgery: Surgical Videos

- *Databases*: Arznei Aktuell, Arzneimittel Pocket 2018, eRef App, Orthorad, ICD-10 Diagnoseauskunft, PRO-IMPLANT Pocket Guide, Pedi Help App, MDCalc Medical Calculator, BoSTT, MRI Essentials, Ortho Guidelines

- *Education*: AMBOSS Wissen für Mediziner

**Apps for rehabilitation**

- Sprunggelenks-App
- DocCheck Help-Arzt

### Future Prospects and Risks for Medical Apps

In addition to information on the usage behavior of the surveyed physicians, this study was able to gain some insights into expected features and risks with regard to future smartphone usage and apps. According to the surveyed physicians, intuitive usability was considered the most important factor for regular use, followed by the quality of apps. However, the development of an intuitive frontend is complex and involves high development costs and test phases [33]. Nevertheless, approximately half (92/201, 45.8%) of the orthopedists and trauma surgeons expected mobile software to be free of charge, and most of the respondents (145/201, 72.1%) favored advertisement-free apps. This raises the question of financing the development of high-quality apps in the future.

Paradoxically, the transparency of the development process was not considered important by the surveyed physicians, indicating the possibility of potential risks stemming from app development being overlooked. Thus, it appears that the risk of using smartphones in clinical/patient practice (ie, alienation from the patient) was underestimated by the study participants who, instead, focused to a greater extent on data misuse and the danger of using untrustworthy apps. In fact, recent data scandals have led to a fundamental distrust of applications that might be implemented in the context of “Big Data” [34].

Artificial intelligence (AI) was of no significance for the majority (176/201, 87.6%) of the respondents, although several already established apps in the medical (and orthopedic) field use AI algorithms. Given that technological progress has already successfully optimized the use of AI in apps and supportive AI systems are being applied in radiology, the interest in mHealth apps geared toward orthopedics and trauma surgery is still quite low [35-38].

Future app usage could offer great benefits in terms of data collection and retrieval; however, their benefits as a medium for improved doctor-patient communication in daily clinical routine are perceived to be low. The possibility of simplified data processing using smartphones is unquestionable, but it could offer a solution for the continuously increasing bureaucratic efforts and limited human resources [39,40].

### Limitations

There are some limitations to this study. The 206 participants of the survey may not be representative of all orthopedics and trauma surgeons in Germany. The subjects in our cohort were younger compared to the average age of DGOU (Society for Orthopaedics and Trauma) members. However, the findings of this survey were consistent with those of a study estimating the use of smartphones in daily clinical practice [41]. The survey primarily addressed general clinicians in maximum care hospitals, leading to a potential bias toward academic centers and younger orthopedic or trauma surgeons. It can be said that smartphone-savvy orthopedics and trauma surgeons primarily participated in our survey. The vision of the “early adopters” can, however, be more informative than nationally representative data in relation to the future potential for smartphone-based benefits in clinical practice. Further studies are also necessary to obtain evidence regarding app usage in the ambulatory sector.

### Outlook

After the initial groundbreaking steps, Germany’s legislature recently provided considerable momentum in the direction of a stringent national digitization strategy in the country. The Law for Better Care Through Digitization and Innovation (Digitale-Versorgung-Gesetz, DVG) passed by the Bundestag on November 7, 2019, paved the way for apps on prescription, the improved use of web-based video consultation services, and greater security in the communication of health data [42]. However, the majority of German orthopedics and trauma surgeons, especially the decision makers, are unfamiliar with the contents of the DVG despite their basic positive interest in digitization. Skepticism currently prevails regarding apps on prescription and potentially unpredictable risks [43]. In a recent joint letter, the German National Association of Statutory Health Insurance Physicians opposed the digitization plans of the Federal Minister of Health. Lack of interface interoperability and the development of software solutions that did not meet the target group’s requirements were identified as major shortcomings [44]. One of the most complex and unsolved problems so far is the integration of health apps into hospital information systems and the upcoming German electronic medical record in 2021. Germany is sorely in need of suitable health apps.

**Figure 5:**
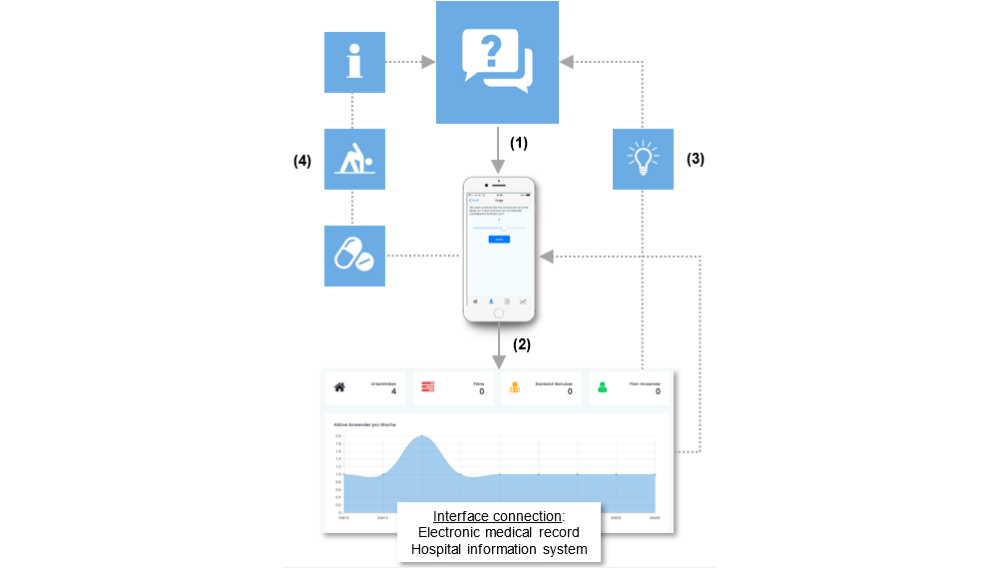
Future innovative usage scenarios of apps in daily clinical routines requiring the implementation of smartphones as the central information and communication medium. (1) Collection of patient-related data via smartphones (wearables). (2) Data storage into databases communicating with additional information systems. (3) Data processing by AI (including determination of risk factors or patterns, conducting interdisciplinary case discussions, and facilitating data backflow to the patient (individualized therapy recommendations and patient monitoring). (4) Treatment recommendations (exchange/communicate information with other physicians; eg, digitized guidelines for antibiotic treatment).

Adequate evaluation of smartphone and app usage requirements is essential for the development and implementation of future innovative digital technologies. However, interface interoperability represents a prerequisite for successful implementation of medical apps (Figure 5). Collaboration between the medical and information technology sectors and the legislature via interdisciplinary expert groups and the involvement of medical societies are essential.

### Conclusions

The study demonstrates that some orthopedics and trauma physicians in Germany already use smartphones and apps on a regular basis in everyday clinical practice. The continued development of apps was considered to provide the greatest future benefit for daily practice. However, some alarming trends are also emerging. If the demand for high-quality apps becomes apparent, the development process is likely to become more complex and cost-intensive. In the context of software development, monetary-, advertising-, or data-based refinancing represent the cornerstones of project realization. Therefore, special attention must be paid to completely plausible transparent financing, development, and data flow for future apps. Currently, discrepancies persist between the supply and demand of orthopedics and trauma surgical apps, creating a legal and ethical vacuum with regard to data protection.

## Abbreviations

AI: artificial intelligence
CE: Conformité Européenne
DGOU: Society for Orthopaedics and Trauma
mHealth: mobile health
